# α_2_δ Modulators for Management of Compression Neuropathic Pain: A Review of Three Case Series

**DOI:** 10.4103/0973-1075.58459

**Published:** 2009

**Authors:** Tariq A Tramboo, Showket Gurkhoo

**Affiliations:** Relief Interventional Pain Clinic, Srinagar, India; 1 Department of Anesthesia and Pain, Shere Kashmir Institute of Medical Sciences, Srinagar, India

**Keywords:** Gabapentin neuropathic pain, Pregabalin, Radiculopathy

## Abstract

**Context:**

The α_2_δ modulators gabapentin and pregabalin are effective against neuropathic pain. Numerous brands of α_2_δ modulators are available in India, and are expected to have equivalent clinical effects. They are routinely used for management of neuropathic pain associated with radiculopathy.

**Aim:**

To describe clinical outcomes in three series of cases of neuropathic pain treated with three available brands of α_2_δ modulators.

**Settings and Design:**

Retrospective analysis of clinical outcomes in patients attending an interventional pain clinic

**Patients and Methods:**

One hundred and ninety-four consecutive patients with neuropathic pain secondary to Magnetic Resonance Imaging (MRI)-documented compression radiculopathy received either LYRICA (LYR), a locally available generic brand of pregabalin (PGN), or a locally available generic brand of gabapentin (GBN), respectively. Drug treatment was continued till adequate pain relief was achieved. In each of the three groups, mean pain scores were analyzed at Days 0, 15, 60 and 90, and daytime sedation scores at Days 1, 15, 60 and 90.

**Statistical Analysis Used:**

Analysis of variance (ANOVA) followed by pair-wise comparison using two-tailed unpaired *t*-test (if *P* value was significant).

**Results:**

Mean pain score was significantly lower in the LYR series as compared to the PGN and GBN series at Days 15, 60 and 90. As compared to the PGN and GBN series, a greater proportion of patients in the LYR series could discontinue drug therapy following adequate pain relief, by Day 90. Daytime sedation scores were significantly lower in the LYR series as compared to the PGN and GBN series at Days 1, 15 and 60, and as compared to the PGN series at Day 90.

**Conclusion:**

These results indicate the effectiveness of α_2_δ modulators for management of neuropathic pain secondary to compression radiculopathy. The results also suggest a possible therapeutic superiority of LYRICA over locally available generic brands of pregabalin and gabapentin. These findings need to be further examined in randomized, controlled trials.

## INTRODUCTION

Pain arising as a result of compression of spinal nerve roots comprises both - nociceptive and neuropathic components. In case of herniated disc compressing a nerve root for example, physiological activation of peripheral nociceptors on the affected nerve root will give rise to nociceptive pain, while ectopic impulse generation in the affected root will give rise to neuropathic pain. Gabapentin and pregabalin, which belong to a new class of drugs referred to as α_2_δ modulators, have been shown to be effective in the management of neuropathic pain associated with multiple conditions including diabetic peripheral neuropathy,[1–4] herpes zoster,[4–7] spinal cord injury[8] and cancer.[9] Given their efficacy in multiple types of neuropathic pain, these drugs are also likely to be effective in neuropathic pain associated with nerve root compression though no randomized controlled study has examined their efficacy specifically in this condition.

Despite a similar mechanism of action, pregabalin offers numerous advantages over gabapentin. It was more potent than gabapentin in pre-clinical studies.[10] Its bioavailability (> 90%) is higher than that of gabapentin (<60%) and unlike gabapentin, is not dose-dependent. Also unlike gabapentin, there is little inter-individual variability in its pharmacokinetics, and blood levels achieved are predictable.[11,12] In clinical studies, pregabalin offered higher responder rates than gabapentin (NNT = 4.2 vs. 5.1).[13] At the same time, it is better tolerated than gabapentin at therapeutic doses (discontinuation due to adverse events = 10.8% vs. 16%).[11,12] In contrast to gabapentin, treatment with pregabalin can be safely initiated at effective doses.[11,12] Unlike gabapentin, pregabalin has demonstrated significant pain relief within three days of starting treatment.[5]

In India, multiple brands of gabapentin and pregabalin are available. LYRICA, the innovator brand of pregabalin, is available at a premium over its generic versions. While NEURONTIN, the innovator brand of gabapentin is not available in India, many of its generic versions are available. Given the substantial price differential between LYRICA and the generic brands of gabapentin and pregabalin, generic prescribing is often adopted as a strategy to contain the treatment cost of neuropathic pain. Since generic brands have the same chemical entity as the innovator brand, and since these brands are required to demonstrate equivalent bioavailability (similar blood concentration profile over time) to the innovator brand, they are expected to have exactly the same clinical effects as the innovator brands.

In the current paper, we report the clinical outcome in three series of cases of neuropathic pain secondary to compression radiculopathy treated with three different brands of α_2_δ modulators namely: LYRICA (LYR), a locally available generic brand of pregabalin (PGN), and a locally available generic brand of gabapentin (GBN), respectively.

## PATIENTS AND METHODS

### Patients

We recruited 194 consecutive patients attending our interventional pain clinic with neuropathic pain secondary to MRI-documented compression radiculopathy without any associated motor involvement. The pain had not been not adequately relieved by prior gabapentin treatment. Those patients who were receiving or were likely to require multiple neuropathic pain medications were excluded. Similarly, patients having concomitant peripheral painful neuropathies were excluded.

### Drug administration

We divided the patients into three series. Sixty-seven patients continued to receive gabapentin, while 62 patients were started on LYRICA, and 65 on generic pregabalin. LYRICA and pregabalin were administered in dosages ranging from 150-600 mg daily, while the target dose for gabapentin was 3000 mg daily. All three drugs were administered in dosages based on patient response and tolerability. We continued treatment till the patients had adequate pain relief, in the event of which, therapy was discontinued.

### Assessment

Pain scores were assessed on an 11-point numerical rating scale (ranging from 0 = ‘no pain’ to 10 = ‘worst possible pain’) on Days 0, 15, 60 and 90, irrespective of whether the patient was still receiving therapy or not. Intensity of daytime sedation experienced by the patients was assessed using a 4-point scale (ranging from 0 = ‘no sedation’ to 3 = maximum sedation') on Days 1, 15, 60 and 90.

### Statistical analysis

Pain as well as daytime sedation scores were analyzed using analysis of variance (ANOVA) followed by pair-wise comparison using two-tailed unpaired *t*-test (if *P* value was significant).

## RESULTS

### Patients

**Figure 1:**
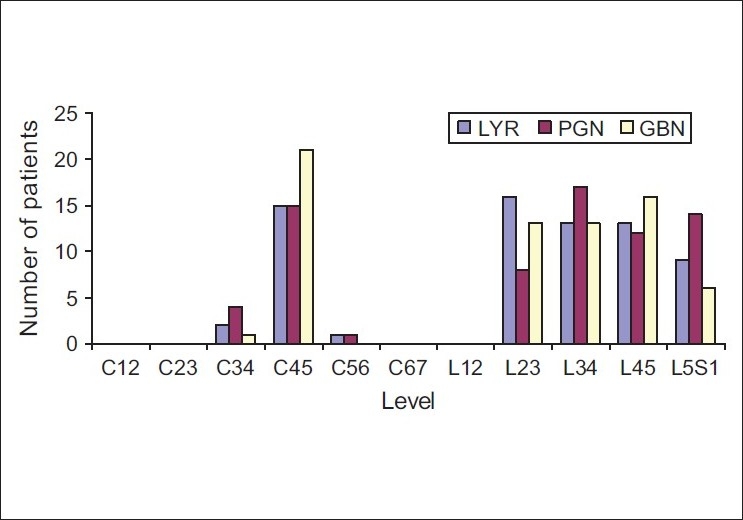
Level of rediculopathy secondary to disc prolapse

Of the 194 patients, the majority (191) had neuropathic pain secondary to radiculopathy associated with disc prolapse, while three patients had radiculopathy associated with canal stenosis. Patient distribution per the level of disc prolapse in each of the three series is depicted in [Figure 1].

### Mean pain scores

**Figure 2:**
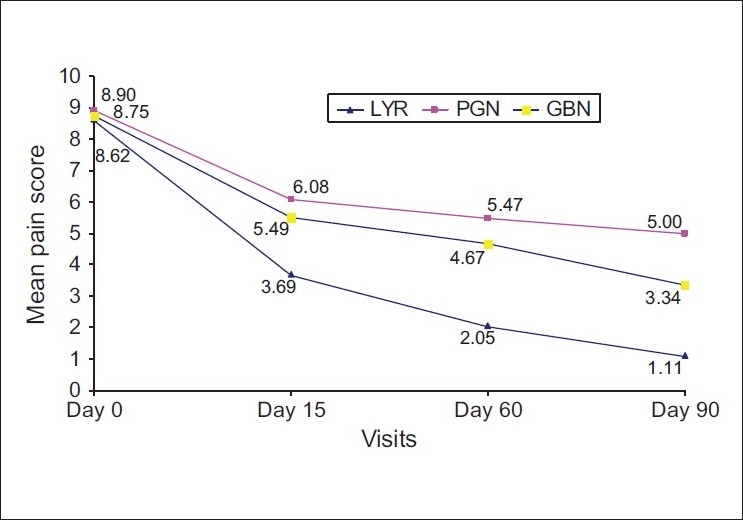
Mean pain scores across visits

An analysis of mean pain scores is summarized in [Table 1], while [Figure 2] graphically depicts the change in these scores over time. Baseline pain scores were equivalent in the three series. However, at Days 15, 60 and 90, the mean pain score in the LYR series was significantly lower than that in the PGN and GBN series. Further, as summarized in Table 2, patients in the LYR series received lower mean drug doses than patients in the PGN series. The mean pain score in the GBN series was significantly lower than that in the PGN series at Days 15, 60 and 90.

**Table 1:** Summary of mean pain score analysis

Day	LYR mean (SE)	PGN mean (SE)	GBN mean (SE)	*P* value		
				
				LYR vs. PGN	LYR vs. GBN	PGN vs. GBN
0	8.62 (0.14)	8.90 (0.14)	8.75 (0.14)	ns	ns	ns
15	3.69 (0.17)	6.08 (0.17)	5.49 (0.16)	<0.0001	<0.0001	0.01
60	2.05 (0.17)	5.47 (0.17)	4.67 (0.17)	<0.0001	<0.0001	0.001
90	1.11 (0.20)	5.00 (0.20)	3.34 (0.19)	<0.0001	<0.0001	<0.0001

**ns: Not signifiacant**

**Table 2:** Mean drug dosages in the three series

Day	Mean drug dose (mg/d)		

	LYR	PGN	GBN
1	258.62	241.53	1800
30	143.53	315.25	1450.82
90	56.9	272.03	877.05

### Duration of drug therapy

Discontinuation due to adverse events was low in all three series. As depicted in Table 3, a greater proportion of patients in the LYR series could discontinue drug therapy following adequate pain relief than in the PGN and GBN series.

**Table 3:** Treatment discontinuations during 90-day follow up

Series	Discontinuation	

	Cause	No. of patients
LYR	Lost to follow-up	2
	Adverse events	1
	Lack of response	1
	Surgical intervention due to intractable pain	0
	Adequate pain relief*	44
PGN	Lost to follow-up	1
	Adverse events	3
	Lack of response	1
	Surgical intervention due to intractable pain	1
	Adequate pain relief*	0
GBN	Lost to follow-up	0
	Adverse events	3
	Lack of response	1
	Surgical intervention due to motor deficit	2
	Adequate pain relief*	10

* Patients who could discontinue drug due to adequate pain relief, and did not need to restart the same during the 90-day period

### Daytime sedation

**Figure 3:**
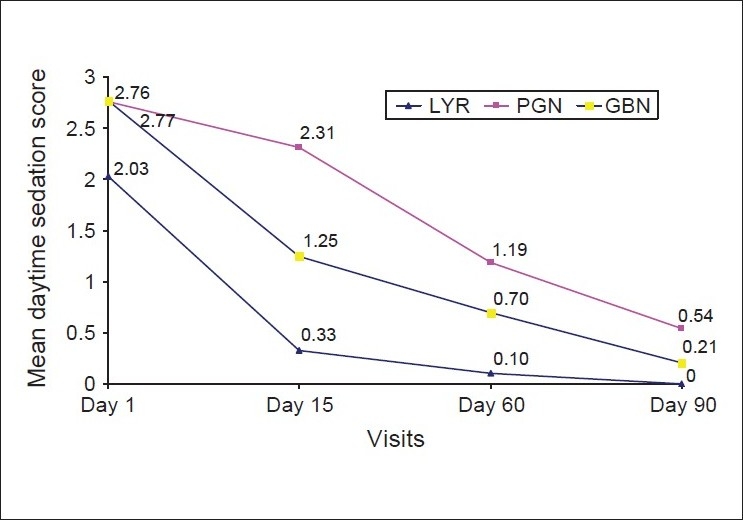
Mean daytime sedation across visits

An analysis of mean daytime sedation scores is summarized in Table 4, while [Figure 3] graphically depicts the change in these scores over time. On Days 1, 15, and 60, daytime sedation scores were significantly lower in the LYR series than in the PGN and GBN series. On Day 90, the scores in the LYR series were significantly lower as compared to the PGN series, but not the GBN series. On Days 15, 60 and 90, daytime sedation scores were significantly lower in the GBN series as compared to the PGN series.

**Table 4:** Summary of mean daytime sedation score analysis

Day	LYR mean (SE)	PGN mean (SE)	GBN mean (SE)	*P* value		
				
				LYR vs. PGN	LYR vs. GBN	PGN vs. GBN
1	2.03 (0.07)	2.76 (0.07)	2.77 (0.07)	<0.0001	<0.0001	ns
15	0.33 (0.11)	2.31 (0.11)	1.25 (0.11)	<0.0001	<0.0001	<0.0001
60	0.10 (0.08)	1.19 (0.08)	0.70 (0.08)	<0.0001	<0.0001	<0.0001
90	0	0.54 (0.08)	0.21 (0.08)	<0.0001	ns	<0.004

ns: Not signifiacant

## DISCUSSION

Our analysis points to the effectiveness of α_2_δ modulators in relieving neuropathic pain secondary to nerve root compression. The results of our analysis also suggest superior efficacy and tolerability for LYRICA as compared to locally available generic brands of pregabalin and gabapentin. Mean pain scores at all time points measured were significantly lower in the LYR series as compared to the PGN and GBN series. Further, lower drug dosages were required in the LYR series as compared to the PGN series. With regards to tolerability, daytime sedation scores were significantly lower in the LYR series as compared to the PGN and GBN series.

Interestingly, the therapeutic outcome in the GBN series was superior to that in the PGN series. All patients in our series had received prior gabapentin, but without adequate pain relief. A decrease in mean pain scores in the GBN series during the study duration can be explained by an increase in the dosage of gabapentin; many patients had earlier received inadequate doses of gabapentin.

Duration of therapy was shorter in the LYR series as compared to the PGN and GBN series; greater proportion of patients in the LYR series had discontinued therapy by Day 90 following adequate pain relief. This could possibly explained by more effective pain relief allowing for greater physical therapy and relief of muscle spasm.

In the past, differences between innovator and generic brands with regards to therapeutic outcome in patients have been reported for other antiepileptic drugs, including phenytoin, carbamazepine and sodium valproate.[14] Though generic brands contain the same chemical entity as the innovator brand, they can differ from the innovator brand in various other aspects like the manufacturing process, purity, enantiomeric ratio, salt of active moiety, or the excepients.[14] Hence there may be differences in the appearance, taste and shelf life of generic and innovator brands, as also clinical differences in therapeutic and adverse effects. Indeed, commentators have noted that bioequivalence need not necessarily guarantee therapeutic equivalence, i.e., it does not guarantee that a drug will have the same therapeutic and adverse effects as the reference drug.[14]

Chemically, pregabalin [s-(+)-isobutyl GABA] is the s-enantiomer of isobutyl GABA. The r-enantiomer of isobutyl GABA lacks activity at α_2_-δ site, and its presence in a pregabalin formulation may be regarded as an impurity.[15] Theoretically it could also possess undesirable activity that could contribute to adverse effects *in vivo*. It is thus, a potential impurity that could arise during synthesis of pregabalin. It is possible that different brands of pregabalin use different manufacturing processes that yield varying proportions of s- and r-enantiomers of isobutyl GABA, thereby explaining the difference in the therapeutic and adverse effects of two different brands of same molecule. This highlights the need for enantio-specific analyses of chiral drugs whose pharmacologic activity resides in one enantiomer.

While the results of our case series suggest a possible therapeutic superiority of LYRICA over generic pregabalin and gabapentin, it will not be appropriate to draw any definite conclusion from the same. We have collected data in our daily outpatient setting and analyzed the same post-hoc, rather than examine the question of the superiority of LYRICA over generic pregabalin or gabapentin in a well-designed clinical study. Hence, the need to confirm the findings of our case series in a formal randomized controlled trial. We hope that our observations will spur further research to address the important issue of therapeutic equivalence of innovator and generic brands of same molecules.
